# Cortical activation and functional connectivity between healthy elderly and Parkinson’s disease patients and between cognitive subgroups of Parkinson’s patients: a multichannel functional near-infrared spectroscopy study

**DOI:** 10.3389/fnagi.2025.1723770

**Published:** 2026-01-09

**Authors:** Xiaodie Liu, Shanshan Zhou, Wenyi Chen, Mengyuan Chen, Yawen Pan, Huabao Xie, Yinghao Zhi

**Affiliations:** 1 Wenzhou TCM Hospital of Zhejiang Chinese Medical University, Wenzhou, China; 2 Department of Rehabilitation Medicine, Wenzhou TCM Hospital of Zhejiang Chinese Medical University, Wenzhou, China; 3 Department of Rehabilitation Medicine, The Second People’s Hospital of Lishui, Lishui, China; 4 Taishun County Traditional Chinese Medicine Hospital, Wenzhou, China

**Keywords:** cognitive impairment, cortical activation, functional connectivity, functional near-infrared spectroscopy, Parkinson’s disease

## Abstract

**Background:**

The rising global burden of Parkinson’s disease (PD) is often related to cognitive decline. Exploring neuroimaging biomarkers is crucial for early diagnosis.

**Methods:**

The purpose of the exploratory research was to look at the differences in cortical activation and functional connectivity between PD patients and healthy controls (HC), as well as among cognitive subgroups of PD, using multichannel functional near-infrared spectroscopy (fNIRS) during a verbal fluency task. A total of 39 PD patients and 20 age-matched HC were assessed.

**Results:**

Results showed significantly reduced oxygenated hemoglobin (oxy-Hb) concentrations in PD patients, particularly in the right temporal lobe, compared to HC. Among PD cognitive subgroups, patients with Parkinson’s disease dementia (PDD) displayed notably lower oxy-Hb levels in key brain regions compared to PD with normal cognition (PD-NC) and PD with mild cognitive impairment (PD-MCI). The analysis among the four groups showed that the HC group and the PDD group had the most differences in activation. Functional connectivity analyses between PD subgroups revealed that PD-NC patients had stronger connectivity between prefrontal regions than PD-MCI and PDD groups.

**Conclusion:**

Our findings generate the hypothesis that PD is associated with altered neurovascular responses and disrupted cortical network organization in the frontal and temporal lobes, especially in cognitively impaired subgroups. These results support the potential utility of fNIRS for characterizing cognition-related neural alterations in PD and provide a basis for future hypothesis-driven and longitudinal investigations.

## 1 Introduction

Over the past 20 years, the incidence and prevalence of Parkinson’s disease (PD) have sharply increased (Deuschl et al., 2020). Motor symptoms include bradykinesia, resting tremor, rigidity, and postural gait abnormalities that are clinically observed in PD sufferers (Bloem et al., 2021). However, numerous PD patients also experience many non-motor symptoms, including a decreased sense of smell, autonomic dysfunction, sleep disturbances, cognitive decline, hallucinations, depression, anxiety, apathy, and pain (Chaudhuri et al., 2006; Tolosa et al., 2021). The disease burden of PD is evolving, resulting in increased disability and decreased functioning for patients and leading to substantial financial costs for families and society (Yang et al., 2020). The early symptoms of PD is more difficult to recognize, and some non-motor symptoms may occur 10–20 years before the diagnosis of PD is made, before clinically significant motor manifestations (Leite Silva et al., 2023).

Cognitive impairment is a core non-motor symptom of PD (Obeso et al., 2017). A variety of cognitive domains are impaired when PD develops, and mild cognitive impairment affects 18.9%–38.2% of individuals with non-demented PD; furthermore, the incidence of mild cognitive impairment in Parkinson’s disease (PD-MCI) patients increases with age, illness progression timeline, and disease severity (Litvan et al., 2011). The duration to the start of dementia varies greatly among individuals with PD, and the risk is high. After receiving a PD diagnosis, approximately half of patients develop dementia within 10 years, and most of them experience Parkinson’s disease dementia (PDD) within 20 years or more (Aarsland et al., 2021). Cognitive impairment greatly impacts patients’ standard of living, functioning, caregiver burden, financial burden and mortality risk (Bäckström et al., 2018; Tang et al., 2020). Clinically, the prominent motor symptoms of PD often cause patients to overlook their cognitive impairment. This lack of awareness can lead to a cascade of secondary issues and makes early diagnosis particularly challenging. Further in-depth research on markers or imaging indicators related to PD cognitive impairment is crucial for the timely identification and intervention of PD cognitive impairment.

Parkinson’s disease is primarily identified through an evaluation of an individual’s clinical history and a detailed neurological assessment (Armstrong and Okun, 2020). Currently, the study of various neurodegenerative diseases, such as PD, also uses imaging techniques, including functional magnetic resonance imaging (fMRI), positron emission tomography (PET), and functional near-infrared spectroscopy (fNIRS), in addition to blood and cerebrospinal fluid markers (Hou and Shang, 2022; Blesa et al., 2023; Schumacher et al., 2023). Early signs of disease are identified by assessing relevant functional connectivity and neurochemical changes to aid in the diagnosis and study of neurodegenerative diseases. The primary function of fNIRS, a non-invasive optical imaging technique, is to identify variations in parameters, including total hemoglobin (total-Hb), deoxyhemoglobin (deoxy-Hb), and oxygenated hemoglobin (oxy-Hb), in brain tissues, thus reflecting the level of brain activity in neurons (Villringer and Chance, 1997). Clinical benefits of fNIRS include security, mobility, non-invasiveness, durability against interference, high comfort, and adaptation compared with other neuroimaging techniques (Rahman et al., 2020). The unique advantages of fNIRS facilitate its sensitive monitoring of cortical region activation and connectivity in PD patients during tasks (Sousani et al., 2023); when combined with neuro-regulation techniques, fNIRS can be used for post-treatment assessment or real-time monitoring during the neuro-stimulation process, thereby promoting the advancement of targeted neuro-regulation therapy (Huo et al., 2023).

This research aimed to examine the similarities and distinctions in cortical activation and functional connectivity between healthy controls (HC) and PD patients during the verbal fluency task (VFT), as well as the discrepancies in these neurophysiological indicators in PD patients with different cognitive levels, and to explore the connections between cortical oxy-Hb concentrations and cognitive performance during this task to further explore early neuroimaging early warning signs of PD.

## 2 Materials and methods

### 2.1 Subjects

39 PD patients were enrolled in the outpatient and inpatient departments of Wenzhou Hospital of Traditional Chinese Medicine between December 2024 and September 2025. Patients with PD had to be (1) aged 20–80 years, (2) fulfilling the diagnostic criteria for Parkinson’s disease in China (2016 version) (Chinese Society of Parkinson’s Disease and Movement and Parkinson’s Disease and Movement Disorder Section of Neurologist Branch of Chinese Medical Doctor Association, 2016), (3) having a revised Hoehn–Yahr classification of stages 1–3, and (4) receiving stable dopaminergic therapy for ≥1 month. These conditions were excluded: (1) secondary parkinsonism or atypical parkinsonism. (2) comorbidities with other major systemic diseases such as liver, kidney, hematology, or immunity; severe psychiatric disorders; and (3) inability to cooperate with the completion of the fNIRS examination or contraindications to the fNIRS examination. 20 age-matched HC without major illnesses were chosen from among the inpatient carers. The study was authorized by the Wenzhou Hospital of Traditional Chinese Medicine Ethics Committee and carried out in compliance with the Declaration of Helsinki (Ethics Registration No. WZY2024-LW-099-01). Everyone who participated provided written informed consent.

### 2.2 Clinical and cognitive assessments

All subjects were interviewed to collect demographic and clinical data. All PD patients were assessed during their medication “on” period in the mid-morning. To minimize rater bias, each patient was independently assessed by two neurologists. The final score was the average of their two ratings. If the two scores differed by more than 10 points, the neurologists re-examined the patient together to reach a consensus. PD patients are clinically staged via the Hoehn and Yahr scale (Hoehn and Yahr, 1967). The overall cognitive performance of PD patients was evaluated with the Parkinson’s Disease-Cognitive Rating Scale (PD-CRS) (Pagonabarraga et al., 2008). The Chinese version of the PD-CRS has good specificity and sensitivity for the cognitive assessment of PD patients (Tan et al., 2020). The total score was 134, the diagnostic cutoff for PD-MCI was 80.5, and the diagnostic cutoff for PDD was 73.5. Group classification was performed using established diagnostic criteria for PD-MCI (Litvan et al., 2012) and PDD (Emre et al., 2007). Based on this, the 39 patients were stratified into the following groups: PD with normal cognition (PD-NC, *n* = 14), PD-MCI (*n* = 13), and PDD (*n* = 12).

### 2.3 Verbal fluency task

**FIGURE 1:**
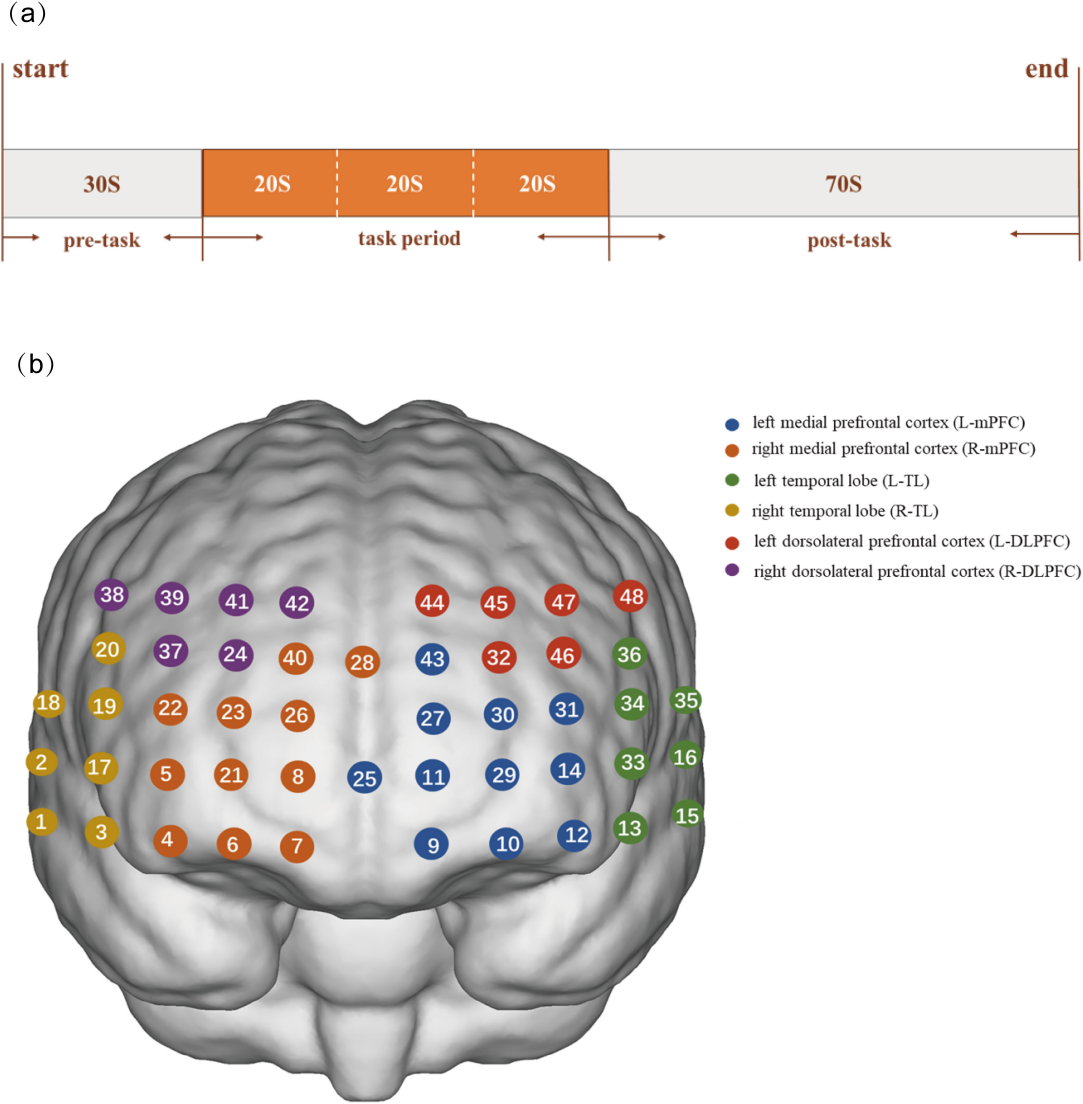
Research task process and channel arrangement diagram. **(a)** Verbal fluency task procedure. **(b)** The detection location.

In this study, VFT task was performed under fNIRS detection. The VFT serves as a standardized neuropsychological instrument for evaluating executive capacity in clinical populations (Aita et al., 2019). The pre-task baseline, task period, and post-task baseline constituted the entire task (Figure 1a). The subjects verbally recited numerals 1 through 5 in sequence in two baseline phases. During the task, the subjects heard three frequently used Chinese characters (“白,” “北,” and “大”) and were instructed to use them to create as many words as they could until they heard the next Chinese character, and the time to form words for each character was 20 s. The primary behavioral outcome was the total number of correct words generated across all three trials, which was recorded for subsequent analysis. Subjects minimized active body movements by sitting in wheelchairs or armchairs with backrests while completing the task. All PD patients underwent fNIRS testing on the day of scale assessment.

### 2.4 fNIRS measurements

In this experiment, hemodynamic changes were recorded via a NirSmart-6000A device (Danyang Huichuang Medical Equipment Co., Ltd., Jiangsu, China) with dual-wavelength (730 and 850 nm) and 11 Hz sampling. The probe array consisted of 15 light-source probes and 16 detection probes, constituting 48 channels (source-detector distance: 2.7–3.3 cm). On the basis of previous studies, it is positioned to cover bilateral prefrontal-temporal cortices with the FPz as the central reference. Six regions of interest (ROIs) were defined: the bilateral dorsolateral prefrontal cortex (L-DLPFC, R-DLPFC), medial prefrontal cortex (L-mPFC, R-mPFC), and temporal lobe (L-TL, R-TL) (Figure 1b; Liu et al., 2022).

### 2.5 fNIRS data quality control and preprocessing

Before the task began, we adjusted the position and pressure of the optical poles to ensure that the original intensity of all channels reached and stabilized within the optimal range recommended by the instrument. During the fNIRS data acquisition, we monitored the raw light intensity oscilloscope provided by the NirSpark platform in real time to ensure the quality of contact. For the areas where the subjects had thick hair, we used a special fiber combing tool to separate the hair to ensure that the optical poles were in vertical and stable contact with the scalp. Following the acquisition of fNIRS signals, the NirSpark platform was employed for hemodynamic data preprocessing. The data preprocessing workflow encompasses the following key stages: (1) use data quality analysis tools to check the data quality and remove the data with poor quality. In this study, the signal-to-noise ratio of channel data was estimated by calculating the coefficient of variation (CV = σ/μ × 100%, where μ is the mean of the signal and σ is the standard deviation of the signal). Higher CV values generally indicate poor signal quality, and for each channel, CV values > 20% were considered bad channels and excluded from further analysis. If more than one-third of the channels in the dataset are judged to be of poor quality, the subject’s data is considered invalid data and excluded from subsequent analysis. (2) The threshold algorithm built into the NirSpark platform was used to automatically detect motion artifacts. For the marked artifact segments, the moving standard deviation and cubic spline interpolation techniques were combined to correct the motion artifacts. For extreme artifacts that cannot be effectively corrected by interpolation, the data of the period or the channel is manually removed. (3) A low-pass filter of 0.2 Hz was used to filter out the high-frequency noise of the instrument, heartbeat, respiration and other physiological noises. (4) Using a modified Beer–Lambert law, the optical density was translated to variations in hemoglobin concentration. The main observation of this investigation was the variation in the mean oxy-Hb concentration detected throughout the VFT, as clinical studies have mostly demonstrated that among fNIRS-derived hemodynamic parameters, oxy-Hb has the highest temporal sensitivity to localized cerebral circulation alterations (Hoshi, 2003). (5) Segmented averaging was performed on VFT blocks within 20–30 s of the pre-task baseline stage, 60 s of the VFT stage, and 110–115 s of the post-task baseline stage. The average change in oxy-Hb concentration for each channel was determined by fitting the two baseline values in a linear fashion. (6) Over the ROIs, the variation in oxy-Hb concentration for every channel was averaged.

### 2.6 Data processing and analysis

SPSS 25.0 was used for statistical analysis. Origin 2021 software and the NirSpark software package were used to generate the graphs and charts. In the case of independent data that met the criteria of normality and homogeneity of variance, parametric analyses employing independent-sample *t*-tests and one-way ANOVA were conducted to evaluate intergroup differences in clinical demographics and oxy-Hb concentration dynamics. The Kruskal–Wallis H test and Mann–Whitney U test were used to evaluate intergroup differences in non-conforming quantitative and ordinal data. To ensure the robustness of the study results and to mitigate the potential impact of confounding variables, age and gender were included as covariates in the statistical model. Univariate analysis of covariance was performed on the normal data of oxygenated hemoglobin concentration in each channel, and ANCOVA analysis was performed on the non-normal data after rank transformation. In the functional connectivity analysis, the functional connectivity value of each ROI-ROI connection was taken as the dependent variable, and a general linear model was constructed with the group as the main independent variable and demographic factors such as gender as the covariate, and the influence of the covariate on the connection strength was statistically controlled. In the activation analysis, the false discovery rate (FDR) correction was used to adjust the *p*-values of multiple channels and brain regions to deal with the problem of multiple comparisons. In the functional connectivity analysis, FDR correction was also used for multiple comparisons of correlation coefficients between all channels. Bonferroni correction was used for *post hoc* comparisons of multiple groups. In addition, Pearson correlation coefficients were used to analyze the correlation between the blood oxygen concentration of the prefrontal cortex (PFC) and the PD-CRS score and VFT behavioral performance to explore the relationship between cortical activity and cognitive level. *Post hoc* statistical power analysis was conducted using G*Power software (version 3.1) based on the observed effect sizes and sample sizes derived from the current dataset. A *p*-value of less than 0.05 was considered statistically significant.

## 3 Results

### 3.1 Group analysis between HC and PD patients

#### 3.1.1 Demographic and clinical characteristics

Table 1 provides a list of the subjects’ demographic details for each group. This investigation involved 59 subjects in total, including 20 in the HC group and 39 in the PD group. Age, education level, and VFT word count were not significantly different between the two groups. Although the differences in the distribution of gender and age between the HC and PD groups did not reach statistical significance, the *p*-value was close to the significant level. Therefore, this study still included gender and age as covariates in the inter-group comparison to control the potential demographic confounding effects.

**TABLE 1:** Demographic and clinical data in the HC and PD groups.

Variable	HC (*n* = 20)	PD (*n* = 39)	*t/Z/*χ ^2^ value	*P*-value
**Age (years)**	66.15 ± 7.400	69.77 ± 7.069	−1.832	0.072
**Gender (*n*)**				
Male	6 (30.0%)	22 (56.4%)	3.698	0.054
Female	14 (70.0%)	17 (43.6%)		
**Education** **(years)**	5.900 ± 4.1536	7.590 ± 4.5924	−1.380	0.173
**VFT correct words**	8.00 ± 3.418	6.38 ± 3.753	1.611	0.113

HC, healthy controls; PD, Parkinson’s disease patients. VFT, the verbal fluency task. The data are presented as the means ± standard deviations.

#### 3.1.2 Changes in mean oxygen saturation during VFT

**FIGURE 2:**
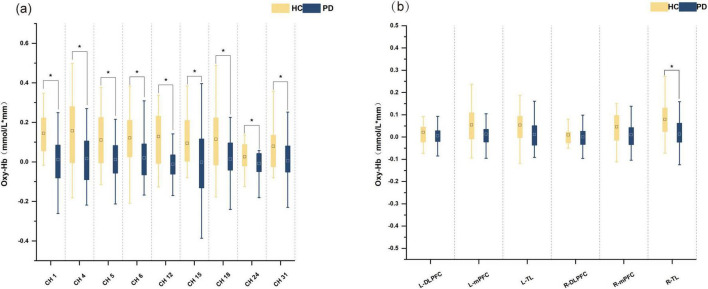
Box plots of oxy-Hb concentrations in the HC and PD groups. **(a)** Oxy-Hb concentrations in channels. **(b)** Oxy-Hb concentrations in various cortical areas. *A significant difference between HC and PD group. ^□^The square represents the mean of the data.

There were notable variations in the mean oxy-Hb concentrations between the two subject groups throughout the task. The HC group had greater mean oxy-Hb concentrations in channels 1, 4, 5, 6, 12, 15, 18, 24 and 31 as compared to the PD group (Figure 2a). In the R-TL, the HC group’s mean oxy-Hb concentrations were substantially greater than the PD group (*F* = 8.115, *p* = 0.006), but the remaining regions of the two groups did not show any discernible variations in the mean oxy-Hb concentrations (Figure 2b).

#### 3.1.3 Changes in functional connectivity during VFT

The mean interchannel connectivity strength was 0.222 (SD = 0.11863) in the HC group and 0.209 (SD = 0.0912) in the PD group. Our study found no significant difference in functional connectivity between the two groups, across either channels or ROIs.

### 3.2 Subgroup analysis of PD

#### 3.2.1 Demographic and clinical characteristics

The demographic differences among the PD subgroups are presented in Table 2. There was a significant variance in the PD-CRS score and VFT word count but no differences in age, sex, education, duration of disease, or H&Y stage among the three groups in the current sample size.

**TABLE 2:** Demographic and clinical data among the PD subgroups.

Variable	PD-NC (*n* = 14)	PD-MCI (*n* = 13)	PDD (*n* = 12)	*F/H/*χ ^2^ value	*P*-value
**Age (years)**	69.57 ± 5.287	68.38 ± 8.977	71.50 ± 6.789	1.421	0.491
**Gender (*n*)**					
Male	10 (71.4%)	7 (53.8%)	5 (41.7%)	2.380	0.304
Female	4 (28.6%)	6 (46.2%)	7 (58.3%)		
**Education (years)**	9.429 ± 3.9945	6.846 ± 4.2787	6.250 ± 5.2071	1.888	0.166
**Disease duration (years)**	6.821 ± 5.4688	4.808 ± 3.3883	5.333 ± 4.7927	1.189	0.552
**H&Y (*n*)**					
I	7 (50.0%)	8 (61.5%)	4 (33.3%)	3.728	0.463
II	5 (35.7%)	2 (15.4%)	6 (50.0%)		
III	2 (14.3%)	3 (23.1%)	2 (16.7%)		
**PD-CRS (score)**	84.71 ± 4.340	76.08 ± 4.716	64.67 ± 7.536	32.543	**0.000**
**VFT correct words**	8.86 ± 2.797	6.00 ± 3.162	3.92 ± 3.728	11.967	**0.003**

PD-NC, Parkinson’s disease normal cognition; PD-MCI, Parkinson’s disease mild cognitive impairment; PDD, Parkinson’s disease dementia; PD-CRS, Parkinson’s Disease-Cognitive Rating Scale. VFT, the verbal fluency task. Bold values represent *p* < 0.05.

#### 3.2.2 Changes in mean oxygen saturation during VFT

**FIGURE 3:**
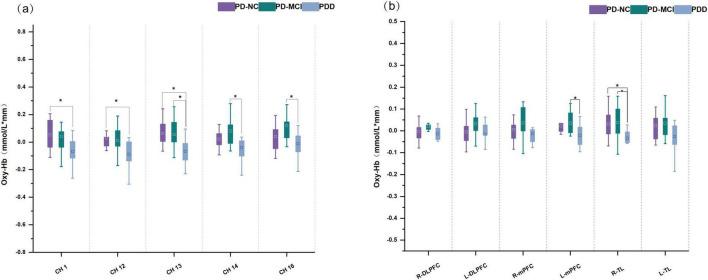
Box plots of oxy-Hb concentrations among PD subgroups. **(a)** Oxy-Hb concentrations in channels. **(b)** Oxy-Hb concentrations in cortical areas. *A significant difference among groups. ^□^The square represents the mean of the data.

Throughout the task, the three groups’ mean oxy-Hb concentrations of the channels varied significantly, with the PDD group’s mean oxy-Hb concentrations being lower than those of the PD-NC and PD-MCI groups. Compared with those in the PD-NC group, the PDD group’s mean oxy-Hb concentrations in channels 1, 12, and 13 were lower; additionally, the PDD group’s mean oxy-Hb concentrations in channels 13, 14, and 16 were significantly lower than those in the PD-MCI group. The mean oxy-Hb concentrations in the PD-NC and PD-MCI groups did not differ significantly (Bonferroni corrected *p* < 0.05; Figure 3a).

The R-TL region (*F* = 5.685, *p* = 0.007) and the L-mPFC region (*F* = 4.943, *p* = 0.013) of ROIs showed statistically significant differences in the mean oxy-Hb concentrations among the three groups of individuals. In particular, the average oxy-Hb concentration in the R-TL region was higher in the PD-NC group than in the PDD group (*adj. p* = 0.042), whereas the PD-MCI group had a significantly greater mean oxy-Hb concentration in the L-mPFC (*adj. p* = 0.012) and R-TL (*adj. p* = 0.013) areas than did the PDD group (Figure 3b).

#### 3.2.3 Changes in functional connectivity during VFT

**FIGURE 4:**
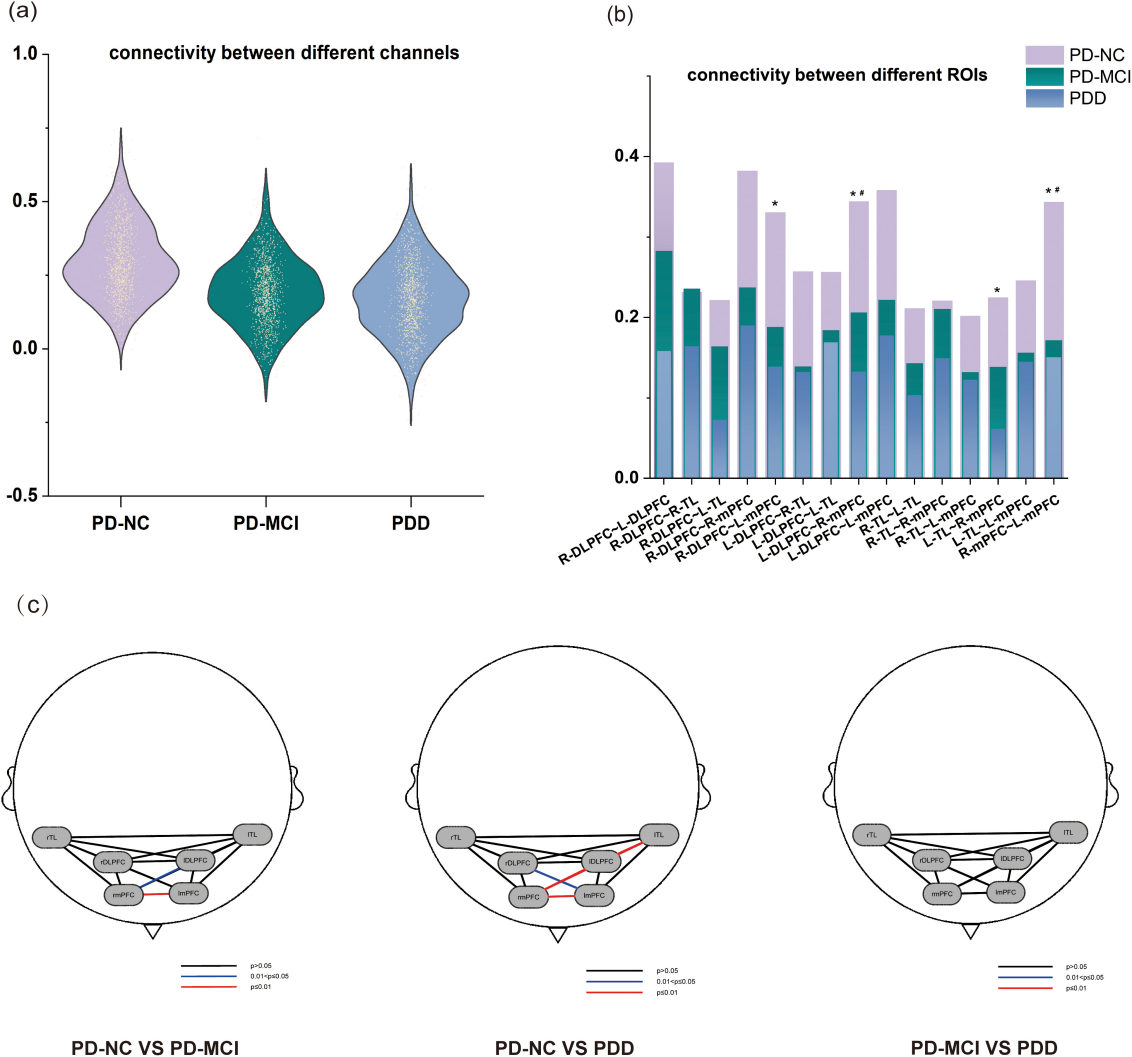
The functional connectivity strength of the PD subgroups. **(a)** Distribution maps of functional connectivity strength among channels in each of the three groups. **(b)** The differences in functional connectivity strength among the ROIs of the three groups. **(c)** Functional Connectivity Strength Maps across ROIs in the Three Groups. *A significant difference between PD-NC and PDD group. ^#^A significant difference between PD-MCI and PDD group.

The mean interchannel connection strength was 0.297 (SD = 0.13226) in the PD-NC group, 0.201 (SD = 0.11525) in the PD-MCI group, and 0.147 (SD = 0.11319) in the PDD group. After inter-ROI connectivity analysis, we concluded that among the three groups of individuals, the PD-NC group’s functional connectivity in the R-mPFC ∼ L-mPFC (*F* = 7.713, *adj. p* = 0.009) and L-DLPFC ∼ R-mPFC (*F* = 6.742, *adj. p* = 0.044) areas were significantly higher than that of the PD-MCI group, and the functional connectivity of the PD-NC group was substantially greater than that of the PDD group in the R-mPFC ∼ L-mPFC (*F* = 7.713, *adj. p* = 0.003), L-DLPFC ∼ R-mPFC (*F* = 6.742, *adj. p* = 0.003), R-DLPFC∼L-mPFC (*F* = 5.092, *adj. p* = 0.020), and L-TL ∼ R-mLPFC (*F* = 5.600, *adj. p* = 0.006) areas. There was no discernible variation in the functional connection strength between the PDD and PD-MCI groups (Figure 4).

#### 3.2.4 Correlation with clinical features

**FIGURE 5:**
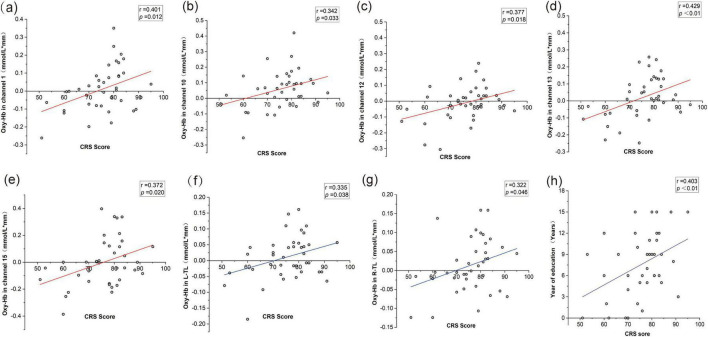
Correlations between PD-CRS scores, oxy-Hb concentrations, and clinical features. **(a)** Correlation between PD-CRS score and oxy-Hb in channel 1. **(b)** Correlation between PD-CRS score and oxy-Hb in channel 10. **(c)** Correlation between PD-CRS score and oxy-Hb in channel 12. **(d)** Correlation between PD-CRS score and oxy-Hb in channel 13. **(e)** Correlation between PD-CRS score and oxy-Hb in channel 15. **(f)** Correlation between PD-CRS score and oxy-Hb in the left temporal lobe (L-TL). **(g)** Correlation between PD-CRS score and oxy-Hb in the right temporal lobe (R-TL). **(h)** Correlation between PD-CRS score and years of education.

A beneficial association with the PD-CRS and the mean oxy-Hb concentration of certain channels was discovered. Channels 1, 10, 12, 13, and 15 were the most prominent ones. The mean oxy-Hb concentrations in both R-TL and L-TL regions were positively correlated with the PD-CRS scores. Furthermore, we also found that the scores were strongly positively correlated with the number of correct words in the patients’ VFT and were correlated with the education level of PD patients (*r* = 0.631, *p* < 0.001), but not with the clinical characteristics of age, the temporal course of the disease, H&Y staging, etc., (see Figures 5).

### 3.3 Group analysis between four groups of subjects

#### 3.3.1 Demographic and clinical characteristics

The demographic characteristics of the four groups of subjects are listed in Table 3. It is evident that the four groups in the current sample size did not differ significantly in terms of age, gender, or educational attainment. However, there were significant differences in the number of VFT correct words among the four groups.

**TABLE 3:** Demographic and clinical data among the four groups.

Variable	HC (*n* = 20)	PD-NC (*n* = 14)	PD-MCI (*n* = 13)	PDD (*n* = 12)	*F/H/*χ ^2^ value	*P*-value
**Age (years)**	66.15 ± 7.400	69.57 ± 5.287	68.38 ± 8.977	71.50 ± 6.789	4.408	0.221
**Gender (*n*)**						
Male	6 (30.0%)	10 (71.4%)	7 (53.8%)	5 (41.7%)	6.045	0.109
Female	14 (70.0%)	4 (28.6%)	6 (46.2%)	7 (58.3%)		
**Education (years)**	5.900 ± 4.1536	9.429 ± 3.9945	6.846 ± 4.2787	6.250 ± 5.2071	4.658	0.199
**VFT correct words**	8.00 ± 3.418	8.86 ± 2.797	6.00 ± 3.162	3.92 ± 3.728	14.796	**0.002**

HC, healthy controls; PD-NC, Parkinson’s disease normal cognition; PD-MCI, Parkinson’s disease mild cognitive impairment; PDD, Parkinson’s disease dementia. VFT, the verbal fluency task. Bold values represent *p* < 0.05.

#### 3.3.2 Changes in mean oxygen saturation during VFT

**FIGURE 6:**
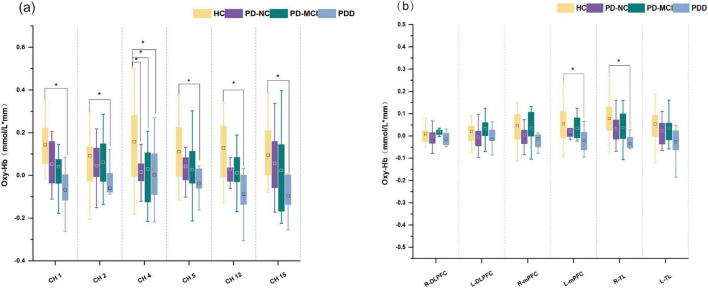
Box plots of oxy-Hb concentrations for four groups. **(a)** Oxy-Hb concentrations in channels. **(b)** Oxy-Hb concentrations in cortical areas. *A significant difference among groups. ^□^The square represents the mean of the data.

**FIGURE 7:**
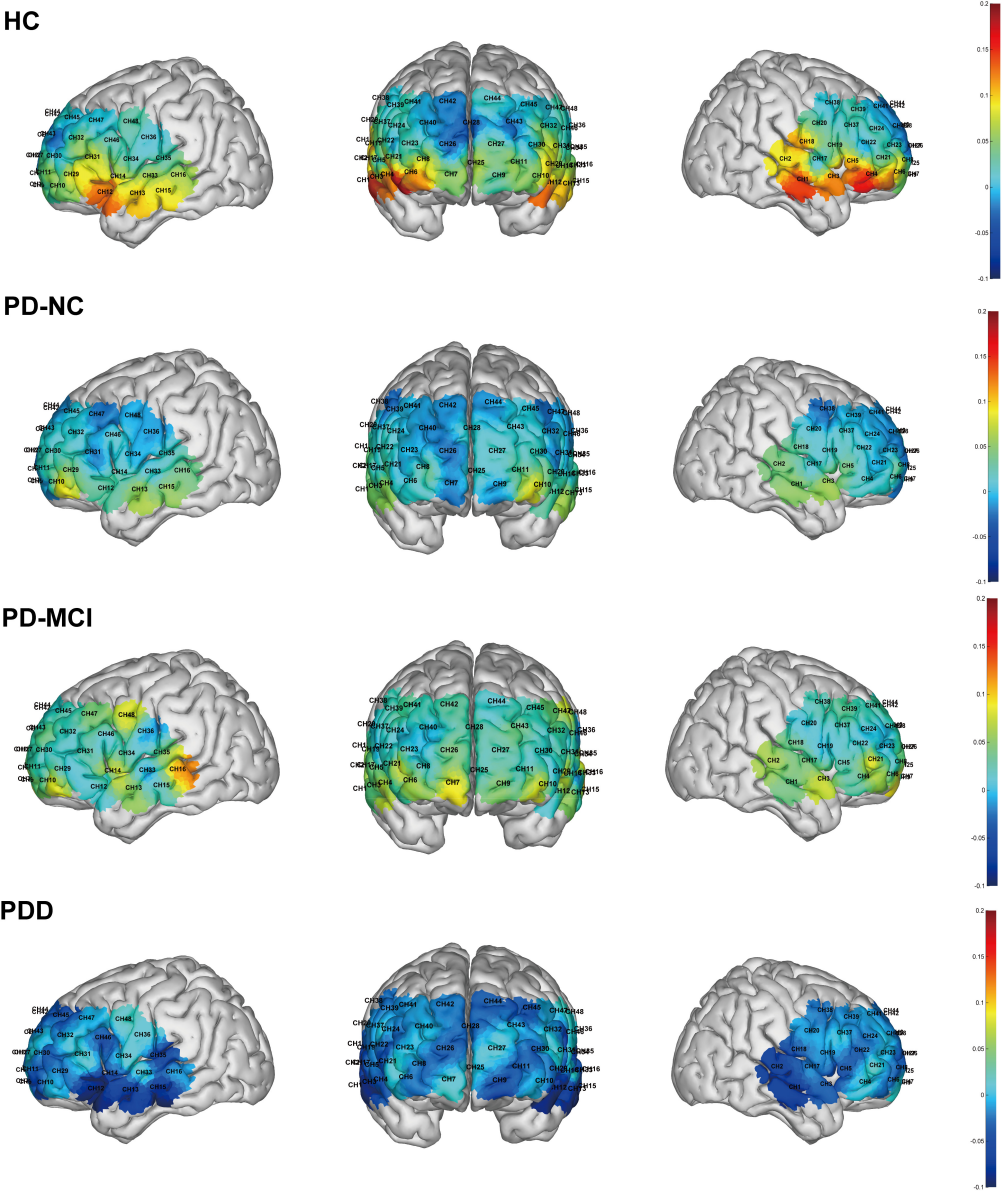
Brain activation maps during the task across the four groups. The color bar indicates the level of activation, with warmer colors corresponding to higher values.

There were significant differences in the mean oxy-Hb concentrations across several channels among the four subject groups during the task. Specifically, the HC group exhibited significantly higher mean oxy-Hb concentration on channel 4 compared to all three PD subgroups. Additionally, compared to the PDD group, the HC group displayed noticeably greater oxy-Hb levels on channels 1, 2, 5, 12, and 15. There were notable variations in the mean oxy-Hb concentrations between the four groups in the R-TL (*F* = 6.530, *p* < 0.001) and L-mPFC (*F* = 3.224, *p* = 0.030) regions within the predetermined ROIs. These changes were especially noticeable between the HC and PDD groups, according to *post hoc* analysis (Bonferroni corrected *p* < 0.05; Figures 6, 7).

#### 3.3.3 Changes in functional connectivity during VFT

After covariate adjustment, the brain functional connection between the R-mPFC ∼ L-mPFC channel showed a significant group main effect among the four groups (*F* = 5.248, *p* = 0.045). *Post hoc* multiple comparisons showed that the difference was mainly between the PD-NC group and the PD-MCI group (*adj. p* = 0.014) and between the PD-NC group and the PDD group (*adj. p* = 0.004).

#### 3.3.4 Correlation with clinical features

In order to explore the relationship between behavioral performance and neural activation and clinical assessment, we performed correlation analysis between VFT performance and the degree of brain activation in the ROI region. The results showed that the number of correct words in VFT was significantly correlated with the activation of the bilateral temporal lobe (R-TL: *r* = 0.390, *p* = 0.014; L-TL: *r* = 0.335, *p* = 0.037), but not significantly correlated with the activation of the remaining ROI regions and demographic variables such as gender, age, and education level.

## 4 Discussion

### 4.1 Cortical activation

The global increase in PD poses a significant public health challenge, particularly due to its strong association with progressive cognitive impairment. Identifying reliable neuroimaging biomarkers has thus become a critical focus for achieving early and accurate diagnosis. Clinical cognitive deficits in PD patients mainly rely on neuropsychological assessment, but clinical assessment is inevitably affected by subjectivity. Currently, a number of neuroimaging techniques are very helpful in determining the brain underpinnings of cognition and cognitive deficits in PD patients (Montaser-Kouhsari et al., 2022), such as structural imaging techniques such as MRI, which help to identify abnormalities in certain brain areas linked to cognitive deficits in PD patients, and fMRI techniques for detecting PD-related cognitive deficits associated with abnormal brain activation and functional connectivity with networks. In this context, the VFT is widely integrated into clinical assessments across diverse populations, including healthy adults and individuals with conditions such as mood disorders, psychiatric illnesses, and neurodegenerative diseases. Its applicability and sensitivity to cognitive changes make it a valuable tool for elucidating neural correlates in PD patients through neuroimaging.

By examining the degree of cortical activation in both groups of subjects under the VFT task, we found that some channels in the prefrontal and temporal cortex were less activated throughout the process in people with PD than in HC individuals; further analysis of the ROIs revealed that PD individuals had considerably reduced mean oxy-Hb concentrations in the R-TL. In one case–control study, Chen et al. (2024) used MRI to scan PD patients and healthy controls. Those authors reported that, in contrast to healthy people, PD patients present widespread gray matter (GM) atrophy in the frontal, temporal, and insula regions of their brains, which may have an impact on their cognitive, motor, and emotional dysfunction (Chen et al., 2024). Another study revealed hypoperfusion and decreased functional connectivity in the occipital region in PD sufferers compared with healthy people, but it failed to detect regional GM atrophy in PD sufferers. Researchers have hypothesized that this could indicate altered neurovascular coupling mechanisms in PD (Laganà et al., 2020). Certain MRI-related studies also point to the presence of extensive cortical perfusion and metabolic decompensation early in the course of PD (Borghammer et al., 2010; Wang et al., 2024). Our hemodynamic analysis showed that the concentration of oxygenated hemoglobin in some cortical regions of PD subjects was lower than that of healthy controls. These findings suggest that there may be changes in neurovascular function in the PD state, which is associated with local cerebral hemodynamic regulation. Based on this preliminary evidence, the multi-channel activation pattern revealed by fNIRS may be used as a potential entry point for future exploration of neurovascular changes in PD, but larger sample and longitudinal design studies are still needed to verify its clinical value.

The results between Parkinson’s subgroups revealed that PD-MCI individuals presented elevated (statistically insignificant) activation in several channels and in the L-mPFC and R-TL regions, whereas PDD individuals’ activity in the relevant regions significantly decreased during the task. In line with the findings of the present study, Yap et al. (2017) clinically reported greater activation in the right prefrontal cortex of MCI patients in a semantic-linguistic fluency task, whereas patients with mild AD showed lower activation. It suggests that patients with MCI have relatively less neurodegeneration and are therefore able to activate neurocompensation to preserve assignment performance, whereas people with AD with neuronal damage have a greater neurocompensatory capacity that may be impaired, leading to the imaging observation of hyperactivity. Although this study, after correction for significance, confirms the role of neurocompensatory mechanisms. The possibility of such a neural compensatory mechanism was also raised in an fMRI study by Yang et al. (2022), which revealed that right angular gyrus activation was increased in the PD-MCI group of patients in a semantic switching task compared with the PD-NC vs. non-PD group, but there was no distinction in neural activity between the groups in a semantic fluency task. The authors suggested that the abnormal activity in the right angular gyrus region might be the result of a potential compensatory mechanism. The semantic switching task is more demanding in terms of executive function than semantic fluency is, causing the right angular gyrus to become more active in order to make up for the cognitive-linguistic deficits experienced by PD-MCI patients, whereas the semantic fluency task is relatively undemanding and thus fails to activate the right angular gyrus region. GM atrophy is more serious and widespread in PDD individuals than in PD-MCI individuals. Compared with HCs and PD patients, PDD patients exhibited considerable frontal and subcortical shrinkage, according to the findings of research on structural and resting-state functional MR changes in the brains of HC, PD, PDD, and DLB patients (Filippi et al., 2020). This structural alteration may lead to greater neuronal damage in areas of atrophic brain regions in patients with PDD, thus exceeding the scope of neural compensation. This mechanism of neural compensation is common in some clinical neurodegenerative diseases. Brain function becomes inefficient or even impaired as a result of pathology, at which point compensatory changes in brain activity can counteract the detrimental effects of neuronal and functional decline and maintain brain performance (Yankner et al., 2008). When brain function degenerates beyond compensation, its role decreases with the progression of neurodegenerative pathology and structural degeneration (Scarmeas et al., 2003). Such changes can often be captured by functional neuroimaging and are manifested as an increase in cerebral perfusion during periods of compensation and a decrease in cerebral perfusion after the failure of the compensatory response. This could also explain the elevated activation of some regions in PD-MCI individuals and the reduced brain activation in PDD individuals compared with PD-NC individuals in the present study.

In the further four-group analysis, we observed that the majority of significant differences were identified between the HC group and the PDD group. This may indicate that the brain activation pattern in the PDD group is substantially distinct from that of both HC and other PD patients with relatively preserved cognitive function. Consistent with this, *post hoc* comparisons of behavioral data also showed that the number of words produced by the PDD group in the VFT was significantly lower than that of the HC group (*p* = 0.017) and the PD-NC group (*p* = 0.003). This indicates that the PDD group not only had obvious behavioral defects, but also had a significant decrease in fNIRS activation in the frontotemporal region. These results together suggest that the PDD subgroup may be the main factor driving the overall difference between PDD and HC, highlighting the importance of stratifying patients according to cognitive status in the study.

### 4.2 Functional connectivity

In the whole-brain analysis, no significant differences in functional connectivity were observed between the HC and PD groups. *Post hoc* power analysis revealed that the effect size of functional connectivity between the ROI regions of the two groups was extremely small, and the statistical power was low, suggesting that the detection ability of functional connectivity differences between HC and PD was limited under the current sample size conditions. This result does not mean that there is no difference between the two groups, but more likely reflects that the observed effect itself is weak, or a larger sample size is needed to be stably detected.

Further considering the disease heterogeneity of PD patients in cognitive performance, we performed subgroup analysis on different cognitive states within patients. The results showed that in the task state, PD-NC patients showed a relatively higher level of functional connectivity than PD cognitive impairment patients. Unlike the overall comparison between HC and PD, the functional connectivity analysis between PD cognitive subgroups showed a medium to large effect size and high statistical power, indicating that the analysis could detect the functional connectivity differences related to different cognitive states more stably under the current research design.

The difference in the results of this analysis suggests that PD patients with different cognitive levels may have different brain functional connections. In PD patients with relatively preserved cognitive function, task-related brain network activity may show a higher level of functional integration, which is consistent with the hypothesis that brain networks may have certain adaptive or compensatory reorganization in the context of the disease. In contrast, in PD patients with a higher degree of cognitive impairment, some specific sub-networks closely related to cognitive processing may show a trend of weakened functional connections. However, given that this study adopts a cross-sectional design, the above explanation is still an exploratory inference and cannot reflect the causal or time series relationship in the disease progression process.

Previous studies based on resting-state functional connectivity have also reported that a progressive decline in cognition in people with PD is linked to alterations in brain connectivity in the resting state (Wang et al., 2021; Delgado-Alvarado et al., 2023). Previous meta-analyses have shown that PD-induced deficits in dopaminergic and cholinergic function, among other factors, can have a wide-ranging impact on brain function, and fMRI can identify clinically discernible reductions in functional connectivity alterations in PD patients with cognitive impairment (Wolters et al., 2019). Brain networks become progressively disrupted, and there are an increasing number of changed connections between brain areas as cognitive impairment in PD progresses (Lopes et al., 2016). Jellinger (2023) reported that PD-MCI patients have substantial functional connection reductions in frontal, temporal, and parietal networks, and in contrast with PD-MCI patients, PDD patients exhibit more widespread functional connectivity alterations that affect almost every functional network associated with attention, memory, general cognition, and other subdomains, suggesting that functions responsible for overall cognition and related generalized failure of brain networks. While the conclusions of the above studies are mostly predicated on functional connectivity analyses during the resting phase, the results of this research point to a potential relationship between PD individuals’ cognitive impairments and frontotemporal lobe functional connectivity during the task state.

To further assess the impact of disease state and cognitive staging relative to the normal baseline at the overall level, we performed a four-group functional connectivity analysis of the HC and PD subgroups. After the four-group comparison analysis, only a few functional connectivity differences between channels were observed, and the significant results were still mainly reflected between the PD cognitive subgroups. After the introduction of healthy controls, the statistical detection ability of the cognitive staging-related differences within PD may be weakened to a certain extent due to the increase in inter-group heterogeneity and overall variance. From a statistical point of view, the network reorganization or compensatory activities that may exist in PD patients with relatively preserved cognitive function may mask the differences between them and healthy controls in the overall PD group analysis.

In addition, in the *post hoc* analysis of the four-group comparison, the effect size of some ROI-to-ROI connections was small, and the statistical power was lower than the conventional 0.80 standard. Although some channel-level connections had high statistical power, their p-values were close to the significance threshold, suggesting that these effects may be more sensitive to sample variability and multiple comparison correction (Supplementary Tables 1–3). In the future, verifying the above results in larger samples and longitudinal designs will help further evaluate the robustness of these functional connectivity changes and their potential significance in the cognitive staging of PD.

### 4.3 Correlation with clinical features

Furthermore, we discovered substantial associations with PD-CRS score and mean oxy-Hb concentrations in the R-TL and L-TL areas, as well as an important positive connection between PD-CRS scores and variations in mean oxy-Hb concentrations in channels 1, 10, 12, 13, and 15. These results suggest that the intensity of neural activity in the temporal lobe region, which is closely related to semantic processing, is not only related to the overall clinical cognitive assessment, but also may be related to the behavioral output of the sensitive cognitive task of VFT. The VFT serves as a sensitive tool for identifying cognitive dysfunction, and frontal and temporal lobe architectures are linked to semantic fluency abilities (Gourovitch et al., 2000). The associations between the R-TL and L-TL regions and cognitive scale scores as well as VFT behavioral performance observed in this study provide further evidence for this clinical trait. However, there was no significant correlation between the PD-CRS score and the activity of the L-mPFC region. Similarly, the VFT performance was not significantly associated with the activation of this region. This possibly because this region initiates more neural compensatory mechanisms to maintain declining brain function in PD-MCI patients, thus affecting the linear correlation between PD-CRS scores and task performance and oxy-Hb concentrations. In addition, the findings may have been limited by the sample size of the study. The sample size of our study was relatively small, and it is difficult to determine whether there was truly no correlation or our study underpowered to find such an association.

The brain functional connectivity and brain activation abnormalities between HC and PD patients and between subjects with different cognitive levels of PD in this study may be studied further in the future as PD markers, offering valuable information for the clinical diagnosis of the disease and its response to treatment.

### 4.4 Limitations

The current research has several significant shortcomings. For our investigation, we first opted for a cross-sectional approach, which revealed varying alterations in brain activation and functional connectivity at various phases of cognitive impairment in PD patients. To determine whether compensatory neuronal responses take place in people with PD-related cognitive impairment over the course of the illness and to look into the threshold and sites where compensatory responses take place, longitudinal fNIRS studies are required. Second, the relatively small sample size represents an important limitation, particularly for subgroup analyses. The limited number of participants likely reduced the statistical power to detect subtle between-group differences and constrained more fine-grained stratification. Although this limitation has been explicitly discussed in the manuscript, future studies will benefit from extended recruitment periods and strengthened multicenter collaboration to increase sample size and improve the robustness and generalizability of the findings. Third, the current study focused exclusively on task-state fNIRS measurements and did not include resting-state assessments of brain activation or functional connectivity. The absence of resting-state data limits direct comparisons with previous neuroimaging studies and precludes a comprehensive evaluation of state-dependent versus task-independent network alterations in PD. Future research incorporating both task-based and resting-state paradigms, along with additional hemodynamic and behavioral measures, may provide a more complete characterization of the neuroimaging correlates of cognitive impairment in PD.

## 5 Conclusion

Our findings suggest that task-related brain activation and functional connectivity patterns differ between PD patients and healthy controls, as well as across PD patients with different levels of cognitive impairment. These observations provide preliminary neurophysiological evidence that may help to characterize disease- and cognition-related neural alterations in PD. While these findings should be regarded as preliminary, they may have potential implications for the future development of neuroimaging markers related to cognitive impairment in PD, pending confirmation in larger and longitudinal studies.

## Data Availability

The original contributions presented in this study are included in this article/Supplementary material, further inquiries can be directed to the corresponding authors.
