# Supplementary material for: Cortical activation and functional connectivity between healthy elderly and Parkinson’s disease patients and between cognitive subgroups of Parkinson’s patients: a multichannel functional near-infrared spectroscopy study
Source: Front Aging Neurosci. 2026 Jan 9;17:1723770. doi: 10.3389/fnagi.2025.1723770 (PMC12827642; doi:10.3389/fnagi.2025.1723770)
Supplement: Supplementary file 1 [file Table_1.docx]

### Supplementary Material

**Supplementary Table1 Post-hoc power analysis of HC and PD groups**

| **Dependent variable** | ***F*** | ***P*-value** | **partial *η²*** | **Cohen’s f** | **Power** |
| --- | --- | --- | --- | --- | --- |
| **Activation analysis** | | | | | |
| Channel 1 | 15.634 | ＜0.001 | 0.225 | 0.599 | 0.982 |
| Channel 4 | 5.776 | 0.020 | 0.097 | 0.328 | 0.697 |
| Channel 5 | 6.863 | 0.012 | **0.117** | 0.364 | 0.784 |
| Channel 6 | 6.195 | 0.016 | 0.106 | 0.344 | 0.738 |
| Channel 12 | 8.627 | 0.005 | 0.138 | 0.400 | 0.855 |
| Channel 15 | 7.860 | 0.007 | 0.127 | 0.381 | 0.820 |
| Channel 18 | 6.411 | 0.014 | 0.110 | 0.352 | 0.757 |
| Channel 24 | 5.028 | 0.029 | 0.085 | 0.305 | 0.634 |
| Channel 31 | 6.034 | 0.017 | 0.101 | 0.380 | 0.715 |
| R-TL | 8.115 | 0.006 | 0.131 | 0.388 | 0.833 |
| **Functional connectivity analysis** | | | | | |
| R-mPFC~L-mPFC | 0.097 | 0.093 | ＜0.001 | 0.027 | 0.051 |
| R-DLPFC~L-mPFC | 0.5014 | 0.6767 | 0.008 | 0.186 | **0.112** |
| L-DLPFC~R-mPFC | 0.9597 | -0.05072 | ＜0.001 | -0.014 | 0.050 |
| L-TL~R-mLPFC | 0.1953 | 0.8459 | ＜0.001 | 0.054 | 0.052 |

**Supplementary Table2 Post-hoc power analysis of PD subgroups**

| **Dependent variable** | ***F*** | ***P*-value** | **partial *η²*** | **Cohen’s f** | **Power** |
| --- | --- | --- | --- | --- | --- |
| **Activation analysis** | | | | | |
| Channel 1 | 4.648 | 0.017 | 0.225 | 0.570 | 0.830 |
| Channel 12 | 6.944 | 0.003 | 0.303 | 0.659 | 0.950 |
| Channel 13 | 5.048 | 0.012 | 0.240 | 0.562 | 0.861 |
| Channel 14 | 4.682 | 0.016 | 0.226 | 0.540 | 0.831 |
| Channel 16 | 4.443 | 0.020 | 0.217 | 0.526 | 0.810 |
| R-TL | 5.685 | 0.007 | 0.251 | 0.579 | 0.882 |
| L-mPFC | 4.943 | 0.013 | 0.225 | 0.539 | 0.830 |
| **Functional connectivity analysis** | | | | | |
| R-mPFC~L-mPFC | 7.713 | 0.024 | 0.312 | 0.674 | 0.959 |
| R-DLPFC~L-mPFC | 5.092 | 0.041 | 0.231 | 0.547 | 0.841 |
| L-DLPFC~R-mPFC | 6.742 | 0.024 | 0.284 | 0.630 | 0.931 |
| L-TL~R-mLPFC | 5.600 | 0.038 | 0.248 | 0.574 | 0.876 |

**Supplementary Table3 Post-hoc power analysis among the four groups**

| **Dependent variable** | ***F*** | ***P*-value** | **partial *η²*** | **Cohen’s f** | **Power** |
| --- | --- | --- | --- | --- | --- |
| **Activation analysis** | | | | | |
| Channel 1 | 5.405 | 0.003 | 0.245 | 0.570 | 0.957 |
| Channel 2 | 2.980 | 0.040 | 0.152 | 0.423 | 0.752 |
| Channel 4 | 3.667 | 0.018 | 0.180 | 0.469 | 0.842 |
| Channel 5 | 3.295 | 0.028 | 0.165 | 0.445 | 0.798 |
| Channel 12 | 3.937 | 0.013 | 0.191 | 0.486 | 0.870 |
| Channel 15 | 3.252 | 0.029 | 0.163 | 0.441 | 0.790 |
| R-TL | 6.531 | ＜0.001 | 0.282 | 0.627 | 0.983 |
| L-mPFC | 3.224 | 0.030 | 0.162 | 0.440 | 0.788 |
| **Functional connectivity analysis** | | | | | |
| R-mPFC~L-mPFC | 5.243 | 0.045 | 0.229 | 0.545 | 0.938 |
| R-DLPFC~L-mPFC | 2.914 | 0.121 | 0.142 | 0.406 | 0.713 |
| L-DLPFC~R-mPFC | 4.437 | 0.055 | 0.200 | 0.501 | 0.890 |
| L-TL~R-mLPFC | 3.403 | 0.119 | 0.162 | 0.439 | 0.786 |
